# Pathological Complete Response and Residual Disease Patterns After Neoadjuvant Chemoradiotherapy in Locally Advanced Esophageal Squamous Cell Carcinoma: Clinical and Laboratory Associations

**DOI:** 10.3390/cancers18182983

**Published:** 2026-09-15

**Authors:** Azer Gökmen, Erdoğan Şeyran

**Affiliations:** Department of Medical Oncology, Van Training and Research Hospital, University of Health Sciences, 65300 Van, Türkiye

**Keywords:** esophageal squamous cell carcinoma, esophagectomy, lactate dehydrogenase, neoadjuvant chemoradiotherapy, pathological complete response, residual disease

## Abstract

**Background:** Pathological response after neoadjuvant chemoradiotherapy for esophageal squamous cell carcinoma may differ between the primary tumor and regional lymph nodes. We evaluated pretreatment factors associated with pathological complete response (pCR) and the anatomical distribution of residual disease after esophagectomy. **Methods:** This retrospective single-center cohort included 100 patients treated with neoadjuvant chemoradiotherapy followed by esophagectomy. pCR was defined as ypT0N0. Associations with pCR were evaluated using logistic regression. **Results:** Among 99 response-evaluable patients, 59 achieved pCR (59.6%; 95% CI, 49.7–68.7%). Of 40 patients with non-pCR, residual disease involved only the primary tumor in 26 (65%), both primary and nodal compartments in 10 (25%), and only regional lymph nodes despite complete primary tumor regression in 4 (10%). Overall, 14 patients with non-pCR (35%) had residual nodal disease. In an exploratory adjusted analysis that could not account for baseline cT and cN categories, lower pretreatment lactate dehydrogenase (OR per 10 U/L increase, 0.93; 95% CI, 0.86–0.99; *p* = 0.026) and female sex (OR, 2.72; 95% CI, 1.02–7.24; *p* = 0.045) were associated with pCR. **Conclusions:** Response was heterogeneous across primary tumor and nodal compartments. Primary tumor regression did not invariably indicate nodal clearance, supporting compartment-specific response assessment. The lactate dehydrogenase and sex associations may reflect residual confounding by baseline disease extent and require external validation.

## Simple Summary

Treatment given before surgery can eliminate esophageal cancer completely in some patients, but the response may differ between the main tumor and nearby lymph nodes. We reviewed 100 patients with locally advanced esophageal squamous cell carcinoma who received chemoradiotherapy followed by surgery. Complete disappearance of cancer from both the esophagus and lymph nodes was observed in approximately 60% of evaluable patients. Among patients with remaining cancer, 35% had residual disease in regional lymph nodes, including a small group in whom the main tumor had completely disappeared. Lower pretreatment lactate dehydrogenase levels and female sex were associated with a greater likelihood of complete response, although these findings require confirmation in larger studies. Our results highlight that disappearance of the main esophageal tumor does not always mean that nearby lymph nodes are free of cancer and support assessment of both locations when evaluating treatment response.

## 1. Introduction

Esophageal cancer is characterized by marked geographical variation in incidence, histological subtype, and clinical outcomes. Esophageal squamous cell carcinoma (ESCC) predominates in many Asian and other high-incidence regions and is often diagnosed at a locally advanced stage because early disease may remain clinically silent. Despite advances in staging, multimodal treatment, and perioperative care, treatment response and clinical outcomes remain heterogeneous, highlighting the need for clinically accessible pretreatment factors that may help characterize the likelihood of pathological response [1,2].

In medically and technically suitable patients with resectable locally advanced disease, treatment commonly integrates neoadjuvant chemoradiotherapy with subsequent esophagectomy [3]. Evidence from the CROSS and NEOCRTEC5010 randomized trials supports this trimodality approach, with improvements in pathological response and survival relative to surgery alone [4,5,6,7]. Nevertheless, pathological response after neoadjuvant treatment varies considerably among individual patients. Pathological complete response requires the absence of viable disease in both the esophageal tumor bed and regional lymph nodes and is therefore classified as ypT0N0. It occurs more frequently in ESCC than in esophageal adenocarcinoma and is associated with favorable disease-control and survival outcomes [8,9]. Residual nodal metastasis in patients with ypT0 disease is associated with poorer outcomes, underscoring the clinical relevance of evaluating residual disease according to both primary tumor and nodal compartments [10,11]. The clinical importance of accurate compartment-specific response assessment has increased further with the emergence of active-surveillance strategies after clinical complete response. The SANO trial met its prespecified non-inferiority criterion for 2-year overall survival when active surveillance was compared with standard surgery in patients with a clinical complete response after neoadjuvant chemoradiotherapy [12]. Nevertheless, the implementation of response-guided surveillance depends on the reliable detection of residual locoregional disease, including nodal involvement that may persist despite complete regression of the primary tumor.

Reliable identification of patients likely to achieve pCR remains challenging. Conventional clinical staging and post-treatment imaging have limited accuracy in distinguishing viable residual tumor from treatment-related inflammation, fibrosis, and necrosis [13]. A recent analysis of the SANO cohort similarly showed that standard clinical parameters had limited ability to predict clinical response after neoadjuvant chemoradiotherapy, further emphasizing the need for improved multimodal response assessment [14]. Although molecular, radiomic, metabolic, and tissue-based biomarkers have been investigated, their routine implementation is constrained by cost, limited availability, methodological heterogeneity, and insufficient external validation. A recent systematic review identified several promising immune and metabolic tumor-microenvironment markers and FDG-PET/CT features, while emphasizing the need for further validation before their routine clinical implementation [15]. In contrast, demographic characteristics and routinely measured pretreatment laboratory variables provide an inexpensive and readily available approach to evaluating host-related and tumor-related factors. Systemic inflammatory indices—including the neutrophil-to-lymphocyte ratio and lymphocyte-to-monocyte ratio—have been examined in relation to treatment response in ESCC, but reported findings have been inconsistent [16,17]. LDH was included among the routinely available laboratory variables because it is an inexpensive and widely measured marker that may integrate several processes potentially relevant to chemoradiotherapy response, including glycolytic tumor metabolism, hypoxia, cellular turnover, tissue injury, and overall disease burden. Although circulating LDH is not tumor-specific, previous studies have demonstrated the prognostic relevance of LDH and LDH-based indices in ESCC; however, its relationship with pathological response after neoadjuvant chemoradiotherapy remains insufficiently characterized [18].

Against this evolving clinical background, a comprehensive evaluation of pathological response requires consideration of both the overall probability of pCR and the anatomical distribution of residual disease. Therefore, this study aimed to evaluate pretreatment clinical and laboratory factors associated with pCR and to characterize residual disease according to primary tumor and regional nodal involvement in patients with locally advanced ESCC undergoing esophagectomy after neoadjuvant chemoradiotherapy.

## 2. Materials and Methods

### 2.1. Study Design and Patient Population

This retrospective, single-center observational cohort study included patients with histologically confirmed locally advanced esophageal squamous cell carcinoma who received neoadjuvant chemoradiotherapy followed by surgical resection at Van Training and Research Hospital between January 2018 and December 2025.

Eligibility as resectable, nonmetastatic, locally advanced disease was based on the contemporaneous multidisciplinary evaluation documented before neoadjuvant treatment. This assessment incorporated upper gastrointestinal endoscopy and available radiological examinations, including contrast-enhanced thoracic and abdominal computed tomography and/or 18F-fluorodeoxyglucose positron emission tomography/computed tomography. Endoscopic ultrasonography was additionally used when available. The multidisciplinary treatment recommendation consisted of neoadjuvant chemoradiotherapy followed by planned surgical resection. Patient-level categorical cT and cN data were not available in the study database and could not be reliably reconstructed from the retrospective records. Consequently, these categories were neither retrospectively inferred nor imputed and could not be summarized or included as covariates in the regression analyses.

Patients were eligible if they had histologically confirmed locally advanced esophageal squamous cell carcinoma, received neoadjuvant chemoradiotherapy, subsequently underwent esophagectomy, and had a postoperative pathology report available in the institutional medical records. Complete ypT and ypN information was not required for inclusion in the overall descriptive cohort but was required for analyses involving pathological response. Patients with a histological subtype other than squamous cell carcinoma, distant metastatic disease at diagnosis, definitive chemoradiotherapy without subsequent surgery, or an unconfirmed neoadjuvant treatment-to-surgery sequence were excluded.

**Figure 1:**
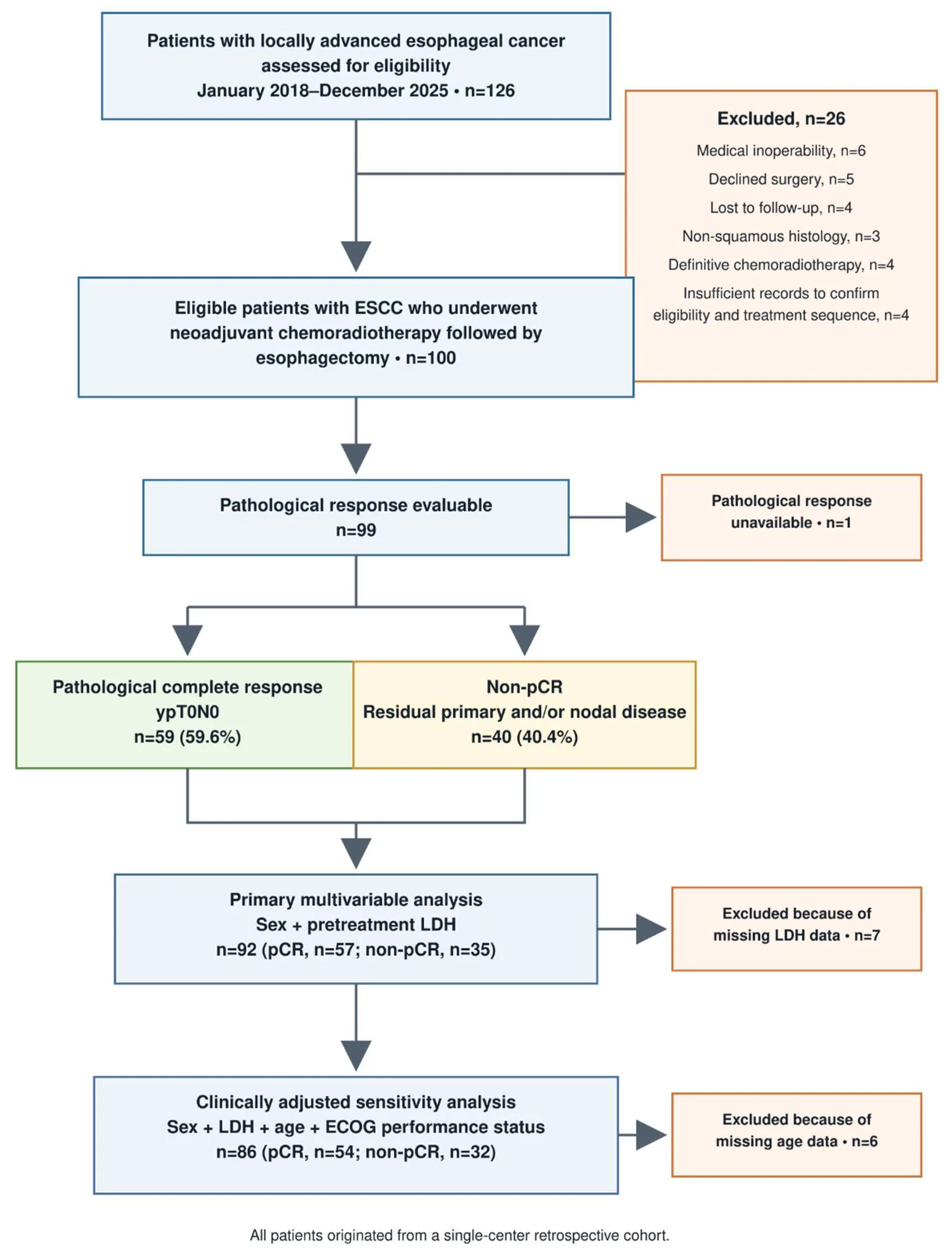
Patient selection and analysis flow diagram. **Figure legend:** Among 126 patients with locally advanced esophageal cancer assessed for eligibility between January 2018 and December 2025, 26 were excluded because of medical inoperability (*n* = 6), refusal of surgery (*n* = 5), loss to follow-up (*n* = 4), non-squamous histology (*n* = 3), receipt of definitive chemoradiotherapy (*n* = 4), or insufficient records to confirm eligibility and the treatment sequence (*n* = 4). The final cohort comprised 100 patients with esophageal squamous cell carcinoma (ESCC) who underwent neoadjuvant chemoradiotherapy followed by esophagectomy. Pathological response was evaluable in 99 patients: 59 (59.6%) achieved pathological complete response (pCR; ypT0N0), whereas 40 (40.4%) had residual primary and/or nodal disease. The primary multivariable analysis included 92 patients with available pathological response and pretreatment lactate dehydrogenase (LDH) data. The clinically adjusted sensitivity analysis included 86 patients with complete data for sex, LDH, age, and Eastern Cooperative Oncology Group (ECOG) performance status.

Demographic, clinical, pretreatment laboratory, and postoperative pathological data were retrospectively obtained from the hospital electronic information system and archived medical records. Patient-level records were reviewed for consistency, and each patient was represented only once in the analytical dataset. During the study period, 126 patients with locally advanced esophageal cancer were assessed for eligibility. Twenty-six patients were excluded because of medical inoperability (*n* = 6), refusal of surgery (*n* = 5), loss to follow-up (*n* = 4), non-squamous histology (*n* = 3), receipt of definitive chemoradiotherapy without subsequent surgery (*n* = 4), or insufficient records to confirm eligibility and the neoadjuvant treatment-to-surgery sequence (*n* = 4). The final study cohort therefore comprised 100 patients with ESCC who underwent neoadjuvant chemoradiotherapy followed by esophagectomy. Pathological response was evaluable in 99 patients; one patient with unavailable ypT and ypN information was retained in the overall descriptive cohort but excluded from analyses requiring classification of pathological response. The study population and the numbers of patients included in the primary and sensitivity analyses are summarized in Figure 1.

### 2.2. Neoadjuvant Treatment, Surgery, and Pathological Assessment

All patients received concurrent neoadjuvant chemoradiotherapy followed by surgical resection with curative intent. Concurrent chemotherapy consisted of weekly paclitaxel 50 mg/m^2^ plus carboplatin AUC 2 in 85 patients (85.0%), cisplatin plus 5-fluorouracil in 7 (7.0%), and other fluoropyrimidine- or platinum-based regimens in 8 (8.0%), according to patient characteristics and institutional practice. Among the 85 patients treated with carboplatin plus paclitaxel, 73 completed all five planned weekly administrations, whereas 8 received four, 3 received three, and 1 received two weekly administrations because of treatment tolerability. Radiotherapy was delivered using intensity-modulated radiotherapy (IMRT), with a planned dose of 41.4 Gy in 23 daily fractions of 1.8 Gy. IMRT was the institutional standard throughout the study period and was selected to provide conformal coverage of the elongated thoracic target volume while limiting radiation exposure to adjacent organs at risk, particularly the lungs, heart, and spinal cord. The choice of technique was protocol-based rather than determined by individual patient or tumor characteristics. Ninety-three patients completed the planned radiotherapy course, whereas seven discontinued radiotherapy before completion because of treatment intolerance. Among these seven patients, the median total delivered radiation dose was 18 Gy (range, 9–34.2 Gy).

Following completion of neoadjuvant chemoradiotherapy, patients were reassessed for operability and distant disease progression. Patients without evidence of distant progression and considered medically suitable for surgery underwent esophagectomy after a median interval of 45 days following completion of chemoradiotherapy. Total esophagectomy was performed in 90 patients (90.0%), whereas 10 (10.0%) underwent subtotal esophagectomy. The extent of esophageal resection was determined according to tumor location and the ability to obtain adequate longitudinal oncological margins. Total esophagectomy was the predominant institutional approach and was favored when required to achieve an adequate longitudinal resection, whereas subtotal esophagectomy was selected in anatomically appropriate cases in which adequate margins could be obtained. Systematic two-field mediastinal and abdominal lymph-node dissection was generally performed, reflecting the longitudinal lymphatic drainage and potential for noncontiguous nodal spread of thoracic ESCC. Cervical lymph-node dissection was added selectively for proximal tumors or suspected cervical nodal involvement. Treatment and surgical decisions were made within a multidisciplinary framework according to institutional practice.

Postoperative surgical specimens were evaluated by institutional pathologists. Resected specimens were formalin-fixed, and the macroscopically identified tumor bed was extensively sampled in accordance with routine institutional pathology practice. Tissue sections were routinely stained with hematoxylin and eosin and examined for viable residual carcinoma. Additional deeper sections and/or immunohistochemical staining were used selectively when routine morphology was equivocal; these procedures were not performed systematically in every case. Routine whole-mount processing and standardized serial step-sectioning of the entire tumor bed were not used. Pathological T and N categories were classified according to the eighth edition of the American Joint Committee on Cancer/Union for International Cancer Control TNM classification. Pathological complete response (pCR) was strictly defined as the absence of viable residual tumor in both the resected primary tumor bed and regional lymph nodes (ypT0N0). Patients with any viable residual primary tumor and/or nodal metastasis were classified as having non-pCR.

Among patients with non-pCR, residual disease was categorized into three mutually exclusive patterns: residual nodal disease only (ypT0N+), residual primary tumor only (ypT + N0), and combined primary and nodal residual disease (ypT + N+). The presence or absence of lymphovascular invasion and perineural invasion was obtained from the postoperative pathology reports. Resection margin status was categorized as R0 or R1/R2 according to the documented pathological assessment. A standardized tumor regression grading system was not routinely documented and was therefore not included in the analysis.

### 2.3. Clinical and Laboratory Variables

Pretreatment demographic and clinical variables included age at diagnosis, sex, Eastern Cooperative Oncology Group (ECOG) performance status, and anatomical tumor location. ECOG performance status was recorded as 0, 1, or 2 and was categorized as 0 versus 1–2 for regression analyses because only a small number of patients had an ECOG performance status of 2. Tumor location was classified as proximal, middle, or distal esophagus and was categorized as distal versus proximal/middle for regression analyses.

Pretreatment laboratory variables obtained within 14 days before the initiation of neoadjuvant chemoradiotherapy included serum C-reactive protein (CRP), lactate dehydrogenase (LDH), albumin, platelet count, absolute neutrophil count, absolute lymphocyte count, and absolute monocyte count. Laboratory values were obtained from the institutional electronic medical records. The institutional reference range for LDH was 125–220 U/L. No outcome-derived cut-off values were used, and laboratory variables were analyzed as continuous measures using clinically interpretable increments.

The neutrophil-to-lymphocyte ratio (NLR) was calculated by dividing the absolute neutrophil count by the absolute lymphocyte count. The lymphocyte-to-monocyte ratio (LMR) was calculated by dividing the absolute lymphocyte count by the absolute monocyte count. The prognostic nutritional index (PNI) was calculated as serum albumin (g/L) + 5 × absolute lymphocyte count (10^9^/L).

For logistic regression analyses, age was modeled per 10-year increase, CRP per 10 mg/L increase, LDH per 10 U/L increase, albumin per 5 g/L increase, platelet count per 50 ×10^3^/µL increase, neutrophil and lymphocyte counts per 1000/µL increase, monocyte count per 100/µL increase, NLR and LMR per one-unit increase, and PNI per five-unit increase. These increments were prespecified to improve the clinical interpretability of the estimated odds ratios.

### 2.4. Study Outcomes

The primary outcome was pathological complete response following neoadjuvant chemoradiotherapy and surgical resection. Pathological complete response was defined as ypT0N0, indicating the absence of viable residual tumor in both the primary tumor bed and regional lymph nodes. Patients with residual primary tumor and/or metastatic regional lymph nodes were classified as having non-pCR.

The primary analytical objective was to evaluate the associations of pretreatment clinical and laboratory variables with the probability of achieving pCR. Adjusted associations were examined using an exploratory parsimonious multivariable model, including sex and pretreatment LDH. This model was intended to estimate adjusted associations rather than to develop a clinical prediction tool.

The secondary objective was to characterize postoperative residual disease among patients who did not achieve pCR. Residual disease was classified as nodal-only residual disease (ypT0N+), primary tumor-only residual disease (ypT + N0), or combined primary and nodal residual disease (ypT + N+). Pathological T and N categories, lymphovascular invasion, perineural invasion, and resection margin status were also descriptively evaluated in the non-pCR subgroup.

### 2.5. Statistical Analysis

Continuous variables were summarized as median and interquartile range because their distributions were non-normal or potentially skewed. Categorical variables were summarized as frequencies and percentages. Percentages were calculated using the number of available observations for each variable. Pretreatment characteristics were compared between the pCR and non-pCR groups using the Mann–Whitney U test for continuous variables. Categorical variables were compared using Pearson’s chi-square test or Fisher’s exact test, as appropriate.

Binary logistic regression was used to evaluate pretreatment factors associated with pathological complete response, with pCR coded as 1 and non-pCR as 0. Univariable models were fitted separately for each demographic, clinical, and laboratory variable. Results were reported as odds ratios (ORs) with 95% confidence intervals (CIs). Continuous predictors were retained on their original continuous scales and expressed using clinically interpretable increments. No outcome-derived cut-off values or data-driven dichotomization procedures were used.

Given the modest sample size and the limited number of non-pCR outcomes, model complexity was intentionally restricted to reduce the risk of overparameterization. The exploratory primary multivariable analysis used a parsimonious association model including sex and pretreatment LDH, the two variables showing evidence of association with pCR in the univariable analyses. Model complexity was evaluated using the conventional threshold of at least 10 outcome events per estimated predictor parameter. With 35 non-pCR outcomes and two predictor parameters in the primary model, the resulting events-per-variable ratio was 17.5. Although the conventional EPV threshold was satisfied and no complete or quasi-complete separation was observed, Firth’s penalized logistic regression was additionally fitted as a sensitivity analysis to evaluate the robustness of the estimates against potential small-sample bias. The Firth model included sex and pretreatment LDH in the same 92-patient complete-case sample as the primary model; profile penalized-likelihood 95% confidence intervals and penalized likelihood-ratio *p*-values were reported. The primary model was based on complete cases with available pathological response and LDH data. Because this parsimonious model involved data-informed variable selection, the robustness of the principal associations was further evaluated in a clinically adjusted sensitivity model additionally including age and ECOG performance status. Multicollinearity was assessed using variance inflation factors and tolerance values. A variance inflation factor greater than 5 or a tolerance value below 0.20 was considered indicative of potentially relevant multicollinearity.

The functional form of the association between pretreatment LDH and pCR was evaluated using restricted cubic spline logistic regression. Knots were placed at the 5th, 35th, 65th, and 95th percentiles of the observed LDH distribution. The spline model was adjusted for sex, and nonlinearity was assessed by comparing the spline and linear LDH models using a likelihood-ratio test. Confidence intervals for the spline-estimated probabilities were obtained using 1000 bootstrap samples. A sex-by-LDH product term was separately evaluated to investigate potential effect modification, and the interaction model was compared with the corresponding main-effects model using a likelihood-ratio test.

As a supplementary assessment of model stability within the study cohort, internal validation of the exploratory sex–LDH model was performed using 1000 bootstrap resamples. Model discrimination was evaluated using the area under the receiver operating characteristic curve, and overall predictive accuracy was assessed using the Brier score. Bootstrap-estimated optimism was used to obtain optimism-corrected values for the area under the curve, Brier score, calibration intercept, and calibration slope. Calibration was additionally examined by comparing predicted probabilities with observed pCR proportions across quintiles of predicted probability.

Potentially influential observations were assessed using Cook’s distance, leverage (hat) values, and DFBETAs. Screening thresholds were defined as Cook’s distance > 4/*n*, leverage > 2*p*/*n*, and an absolute DFBETA > 2/√*n*, where *n* represents the analysis sample size and *p* represents the number of model parameters, including the intercept. These thresholds were used to identify potentially influential observations for sensitivity analysis rather than as automatic criteria for case deletion. The primary model was refitted after excluding observations with Cook’s distance > 4/*n*. Robustness was additionally evaluated after excluding observations with LDH values above the 95th percentile. An additional sensitivity analysis modeled LDH on the log2 scale, with the resulting OR representing the change in the odds of pCR associated with a doubling of LDH.

No formal sample-size calculation was performed because all eligible patients treated during the predefined study period were included. Analyses were conducted using available-case data for descriptive and univariable analyses and complete-case data for each multivariable model. The availability of each variable was reported, and patients included in and excluded from the primary model were compared to assess potential selection related to missing data. Multiple imputation was not performed because the number of patients with missing LDH data was small and the availability of suitable auxiliary variables was limited. Given the exploratory nature of the study, no adjustment was made for multiple comparisons, and secondary analyses were interpreted cautiously. Accordingly, the findings were considered hypothesis-generating, with emphasis placed on effect estimates and 95% confidence intervals rather than statistical significance alone. Wilson 95% confidence intervals were calculated for the principal residual-disease proportions. All tests were two-sided, and *p*-values below 0.05 were considered statistically significant. Statistical analyses were performed using Python version 3.12.

### 2.6. Ethical Considerations

This study was conducted in accordance with the principles of the Declaration of Helsinki and was approved by the Health Sciences University Van Training and Research Hospital Non-Interventional Clinical Research Ethics Committee (approval number: GOKAEK/2026-05-10; approval date: 8 May 2026). Given the retrospective, non-interventional design and the use of anonymized patient data, the requirement for individual informed consent was waived by the Ethics Committee. All study data were handled in accordance with institutional requirements for patient confidentiality and data protection.

## 3. Results

### 3.1. Study Population and Baseline Characteristics

Among 126 patients with locally advanced esophageal cancer assessed for eligibility, 26 were excluded, resulting in a final surgically treated cohort of 100 patients with ESCC who underwent neoadjuvant chemoradiotherapy followed by esophagectomy. Pathological response was evaluable in 99 patients; 1 patient with unavailable ypT and ypN data could not be classified according to pathological response. Among the 99 evaluable patients, 59 achieved pathological complete response (59.6%; 95% CI, 49.7–68.7%), whereas 40 had residual primary and/or nodal disease (40.4%).

The median age was 56.0 years (interquartile range [IQR], 50.2–62.0), based on 86 patients with available age data. Of the 100 patients, 57 (57.0%) were male and 43 (43.0%) were female. ECOG performance status was 0 in 26 patients (26.0%), 1 in 72 (72.0%), and 2 in 2 (2.0%). Tumor location was available in 86 patients and was proximal in 6 (7.0%), middle in 30 (34.9%), and distal in 50 (58.1%).

The median pretreatment LDH level was 182 U/L (IQR, 156–208), the median CRP level was 3.3 mg/L (IQR, 3.0–10.9), and the median serum albumin level was 39.2 g/L (IQR, 34.0–43.0). The median NLR, LMR, and PNI values were 2.25 (IQR, 1.80–2.99), 3.98 (IQR, 2.89–5.10), and 48.4 (IQR, 41.4–54.0), respectively.

Among the 99 patients with evaluable postoperative pathological staging, 63 (63.6%) had ypT0 disease and 85 (85.9%) had ypN0 disease. Lymphovascular invasion was present in 7 of 97 evaluable patients (7.2%), and perineural invasion was present in 13 of 97 (13.4%). Resection margin status was available in 86 patients, of whom 83 (96.5%) had an R0 resection and 3 (3.5%) had an R1/R2 resection. The complete demographic, clinical, laboratory, and pathological characteristics of the cohort are presented in Table 1.

### 3.2. Comparison of Pretreatment Characteristics Between the pCR and Non-pCR Groups

Pretreatment LDH levels were significantly lower among patients who achieved pCR than among those with non-pCR (median, 165 U/L [IQR, 147–194] vs. 194 U/L [IQR, 177–238], respectively; *p* < 0.001). The distribution of sex also differed significantly between the groups (*p* = 0.013). Female patients accounted for 52.5% of the pCR group and 27.5% of the non-pCR group, whereas male patients accounted for 47.5% and 72.5% of the respective groups. The total number of resected lymph nodes was available for 97 patients. Median nodal yield did not differ significantly between the pCR and non-pCR groups: 12 (IQR, 8–15; *n* = 57) versus 13 (IQR, 8.75–17.25; *n* = 40), respectively (Mann–Whitney U = 998; *p* = 0.299).

No significant between-group differences were observed for age, ECOG performance status, tumor location, CRP, albumin, platelet count, neutrophil count, lymphocyte count, monocyte count, NLR, LMR, or PNI (all *p* > 0.05). Detailed comparisons of pretreatment clinical and laboratory characteristics according to pathological response are presented in Table 2.

### 3.3. Univariable and Multivariable Analyses of Pathological Complete Response

In univariable logistic regression analysis, female sex was associated with higher odds of achieving pCR compared with male sex (OR, 2.92; 95% CI, 1.23–6.91; *p* = 0.015). Higher pretreatment LDH was associated with lower odds of pCR, with an OR of 0.91 for each 10 U/L increase (95% CI, 0.86–0.98; *p* = 0.008). None of the other evaluated pretreatment clinical or laboratory variables showed a statistically significant association with pCR.

The primary multivariable model included sex and pretreatment LDH and was based on 92 patients with available data for pathological response and pretreatment LDH, including 57 patients with pCR and 35 with non-pCR. After mutual adjustment, female sex remained associated with higher odds of pCR after adjustment for pretreatment LDH (adjusted OR, 2.72; 95% CI, 1.02–7.24; *p* = 0.045). Higher pretreatment LDH also remained associated with lower odds of pCR after adjustment for sex (adjusted OR per 10 U/L increase, 0.93; 95% CI, 0.86–0.99; *p* = 0.026), corresponding to an approximately 7% reduction in the odds of pCR for every 10 U/L increase in LDH.

**Figure 2:**
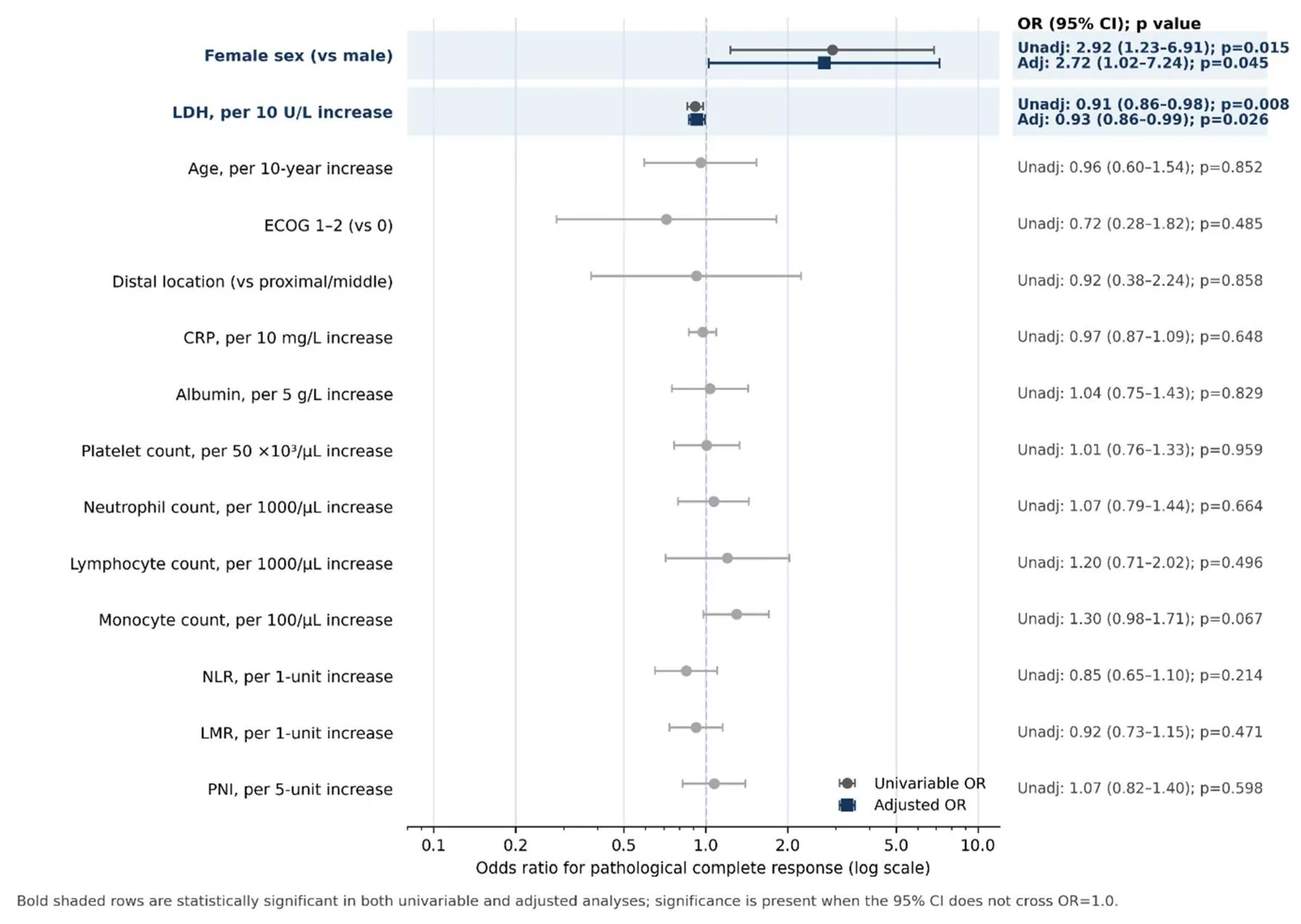
Univariable and multivariable associations of pretreatment factors with pathological complete response. **Figure legend:** Gray circles represent univariable odds ratios, and dark-blue squares represent adjusted odds ratios from the primary multivariable model, including sex and pretreatment LDH. Horizontal lines indicate 95% confidence intervals. The dashed vertical line represents the null value of OR = 1.0. Statistically significant variables in both the univariable and adjusted analyses are highlighted by shaded rows and bold text. Odds ratios are displayed on a logarithmic scale. Pathological complete response was defined as ypT0N0.

The complete univariable and multivariable regression results are presented in Table 3, and the corresponding effect estimates are displayed in Figure 2.

### 3.4. Residual Pathological Disease Patterns

Among the 40 patients who did not achieve pCR, residual primary tumor without nodal involvement (ypT + N0) was the most frequent residual disease pattern and was observed in 26 patients (65.0%). Combined residual primary and nodal disease (ypT + N+) was present in 10 patients (25.0%), while 4 patients (10.0%) had residual nodal disease despite complete regression of the primary tumor (ypT0N+). Overall, residual regional nodal disease was present in 14 of the 40 patients with non-pCR (35.0%; 95% CI, 22.1–50.5%), including 4 patients with ypT0N+ disease (10.0%; 95% CI, 4.0–23.1%).

Within the non-pCR group, the distribution of pathological T categories was ypT0 in four patients (10.0%), ypT1 in four (10.0%), ypT2 in fifteen (37.5%), ypT3 in sixteen (40.0%), and ypT4 in one (2.5%). Twenty-six patients (65.0%) had ypN0 disease, twelve (30.0%) had ypN1 disease, and two (5.0%) had ypN2 disease; no patient had ypN3 disease.

Lymphovascular invasion was present in 7 of 39 evaluable patients with non-pCR (17.9%), and perineural invasion was present in 13 of 39 (33.3%). Resection margin status was available for 32 patients in this subgroup, of whom 29 (90.6%) had an R0 resection and 3 (9.4%) had an R1/R2 resection. Residual pathological disease characteristics are summarized in Table 4.

### 3.5. Sensitivity and Influence Analyses

In the clinically adjusted sensitivity model, which additionally included age and ECOG performance status, the associations of female sex and pretreatment LDH with pCR remained consistent. Female sex was associated with higher odds of pCR (adjusted OR, 3.53; 95% CI, 1.21–10.31; *p* = 0.021), whereas higher LDH was associated with lower odds of pCR (adjusted OR per 10 U/L increase, 0.930; 95% CI, 0.866–0.999; *p* = 0.046). Age (adjusted OR per 10-year increase, 0.86; 95% CI, 0.42–1.76; *p* = 0.685) and ECOG performance status 1–2 versus 0 (adjusted OR, 1.07; 95% CI, 0.25–4.49; *p* = 0.927) were not significantly associated with pCR. The clinically adjusted sensitivity analysis is presented in Supplementary Table S3.

Restricted cubic spline analysis provided no evidence of a nonlinear association between pretreatment LDH and the log odds of pCR after adjustment for sex (*p* for nonlinearity = 0.685), supporting the use of LDH as a continuous linear predictor in the primary model (Supplementary Figure S1). There was also no evidence that the association between LDH and pCR differed according to sex. The sex-by-LDH interaction term was not statistically significant (interaction OR per 10 U/L increase in LDH, 1.090; 95% CI, 0.911–1.304; *p* = 0.347), and comparison of the interaction and main-effects models yielded a likelihood-ratio *p* value of 0.331.

No relevant multicollinearity was detected in either the primary or clinically adjusted model, with all variance inflation factors ≤ 1.64. The maximum Cook’s distance was 0.163, and four observations exceeded the prespecified screening threshold of 4/*n*. Several observations were also flagged by leverage or DFBETA screening; these observations were retained because the thresholds were used for diagnostic assessment rather than as automatic deletion criteria. After exclusion of the four observations flagged by Cook’s distance, the direction and statistical significance of both associations were maintained: female sex remained associated with higher odds of pCR (adjusted OR, 3.04; 95% CI, 1.05–8.81; *p* = 0.041), whereas higher LDH remained associated with lower odds of pCR (adjusted OR per 10 U/L increase, 0.81; 95% CI, 0.70–0.94; *p* = 0.005).

After excluding LDH values above the 95th percentile, the association between higher LDH and lower odds of pCR remained directionally consistent but was no longer statistically significant (adjusted OR per 10 U/L increase, 0.92; 95% CI, 0.83–1.01; *p* = 0.071). When LDH was modeled on the log2 scale, each doubling of LDH was associated with lower odds of pCR (adjusted OR, 0.26; 95% CI, 0.08–0.79; *p* = 0.018).

As an additional sensitivity analysis addressing potential small-sample bias, Firth’s penalized logistic regression yielded estimates consistent with those of the primary model. Female sex remained associated with higher odds of pCR (Firth-penalized OR, 2.60; 95% profile penalized-likelihood CI, 1.03–7.02; *p* = 0.044), whereas higher pretreatment LDH remained associated with lower odds of pCR (Firth-penalized OR per 10 U/L increase, 0.932; 95% profile penalized-likelihood CI, 0.866–0.988; *p* = 0.017).

### 3.6. Internal Model Assessment

The apparent area under the receiver operating characteristic curve of the primary sex–LDH model was 0.726. After internal validation with 1000 bootstrap resamples, the estimated optimism was 0.011, resulting in an optimism-corrected area under the curve of 0.715. The apparent Brier score was 0.202, with an optimism-corrected value of 0.215.

The apparent calibration intercept and slope were 0.000 and 1.000, respectively. After bootstrap correction, the calibration intercept was 0.027, and the calibration slope was 0.944. These findings suggested limited apparent overfitting within the study cohort. However, calibration estimates were interpreted as exploratory because of the modest sample size and the absence of external validation.

The complete bootstrap internal validation results are presented in Supplementary Table S2, and the calibration findings are illustrated in Supplementary Figure S2.

### 3.7. Missing Data and Analysis Populations

Data availability varied across individual clinical, laboratory, and pathological variables. Pathological response was available for 99 of the 100 patients, while pretreatment LDH was available for 92. The primary multivariable model therefore included 92 patients with available pathological response and pretreatment LDH data. Eight patients were excluded from the primary model: seven had evaluable pathological response but lacked pretreatment LDH data, and one lacked both pathological response and LDH information.

The clinically adjusted sensitivity model included 86 patients with complete data for pathological response, LDH, sex, age, and ECOG performance status. No imputation was performed, and the denominators used in descriptive and comparative analyses reflected the number of available observations for each variable.

Patients included in and excluded from the primary model did not differ significantly with respect to sex, ECOG performance status, pathological complete response, ypT0 status, ypN-positive disease, lymphovascular invasion, or perineural invasion (all *p* > 0.05). Nevertheless, female patients constituted 75.0% of those excluded from the primary model compared with 40.2% of those included, although this difference did not reach statistical significance (*p* = 0.072). Comparisons involving the excluded group were based on small and variable denominators and were therefore interpreted cautiously. Detailed variable availability and comparisons according to primary-model inclusion are presented in Supplementary Table S1.

## 4. Discussion

This study demonstrated substantial compartment-specific heterogeneity in pathological response among surgically treated patients with locally advanced ESCC who received neoadjuvant chemoradiotherapy. Although 59.6% of response-evaluable patients achieved ypT0N0, 35.0% of patients with non-pCR had persistent regional nodal disease, including a clinically relevant subgroup with ypT0N+ disease despite complete regression of the primary tumor. Lower pretreatment LDH and female sex were associated with higher odds of pCR in adjusted analyses; however, these exploratory associations cannot be considered independent of baseline disease extent and may partly or entirely reflect unmeasured differences in initial tumor and nodal burden. Collectively, the findings indicate that routinely available pretreatment factors may help characterize response at the cohort level, whereas primary tumor regression alone does not reliably establish nodal clearance.

The pCR rate of 59.6% observed in our cohort was higher than the pooled rate of 32% reported for ESCC in a recent systematic review, although the rates across the included studies ranged widely from 8% to 66% [8]. The relatively high pCR rate in our study should be interpreted in the context of its exclusively squamous histology because ESCC generally demonstrates greater sensitivity to chemoradiotherapy than esophageal adenocarcinoma. In the CROSS trial, pCR was achieved in 49% of patients with squamous cell carcinoma, while the NEOCRTEC5010 trial reported a pCR rate of approximately 43% [4,6]. The 2024 JCOG1109 NExT phase III trial further demonstrated that pathological response rates in locally advanced ESCC vary according to the neoadjuvant regimen, supporting treatment-related heterogeneity across cohorts [19]. Western surgical series have likewise demonstrated substantial pathological downstaging after neoadjuvant chemoradiotherapy and consistently favorable outcomes among patients achieving pCR [20,21]. Several cohort-specific factors may have contributed to the comparatively high pCR rate. Most importantly, this retrospective analysis was restricted to patients who successfully proceeded to esophagectomy after NACRT. Patients who became medically inoperable, refused surgery, were lost to follow-up, received definitive chemoradiotherapy without subsequent surgery, or had insufficient records to confirm eligibility and treatment sequence were excluded. Moreover, patients were reassessed before surgery, and only those without distant progression and considered medically suitable underwent resection. This surgery-only selection may have enriched the cohort for patients with favorable treatment tolerance and operability. Differences in baseline disease extent and other case-mix characteristics may also have contributed; however, detailed baseline cT and cN categories were unavailable and could not be evaluated. The modest sample size additionally permits random variation in the observed response proportion. Regarding surgical factors, total esophagectomy was performed in 90% of patients, generally with systematic two-field lymph-node dissection. The median nodal yield was 12, with no significant difference between the pCR and non-pCR groups. Contemporary surgical guidelines recognize the extensive longitudinal nodal spread of thoracic esophageal cancer and recommend tailoring the extent of esophageal resection and lymphadenectomy according to tumor location, disease extent, patient condition, and the ability to achieve adequate oncological margins [22]. These surgical procedures enabled pathological evaluation of both the primary tumor bed and regional lymph nodes but would not themselves be expected to increase the biological probability of pCR. If anything, systematic nodal assessment reduces the likelihood of incorrectly classifying residual nodal disease as ypT0N0. Therefore, the observed pCR rate should be interpreted as a finding of a selected surgical cohort and should not be extrapolated to all patients initiating NACRT.

Pretreatment LDH was the laboratory variable showing the most consistent directional association with pathological response across the primary and sensitivity analyses. Patients who achieved pCR had substantially lower LDH levels than those with residual disease, and each 10 U/L increase in LDH was associated with an approximately 7% reduction in the adjusted odds of pCR. Although LDH lacks tumor specificity, its circulating concentration may integrate several processes relevant to treatment response, including cellular turnover, tissue injury, hypoxia-associated metabolism, and overall disease burden. Elevated LDH may therefore identify tumors with a more aggressive or metabolically unfavorable phenotype and a reduced likelihood of complete eradication by chemoradiotherapy. Previous evidence has supported the prognostic relevance of LDH-based indices in surgically treated patients with esophageal cancer [18], whereas evidence specifically examining the association between pretreatment LDH and pCR after neoadjuvant chemoradiotherapy remains limited. Our findings support further investigation of LDH in relation to pathological response; however, the observed association may reflect unmeasured baseline disease extent and requires confirmation in an independent cohort.

Several aspects of the LDH analyses support a cautious but coherent interpretation. Restricted cubic spline analysis provided no evidence of departure from linearity, supporting the analysis of LDH as a continuous variable without imposing an outcome-derived threshold. The direction of the association was also preserved when LDH was modeled on the log2 scale. However, after exclusion of values above the 95th percentile, the association remained directionally consistent but no longer reached statistical significance, indicating that observations in the upper LDH distribution contributed to the magnitude and precision of the primary estimate. This finding does not invalidate the observed association but emphasizes the need for confirmation in a larger, independently assembled cohort. Although the absence of a data-derived cutoff limits immediate bedside classification, it reduces the risk of overoptimistic results arising from threshold selection.

Female sex was associated with higher odds of pCR after adjustment for LDH, although the estimate was imprecise, with a wide confidence interval and a lower confidence limit close to the null value. The direction of the association was maintained after additional adjustment for age and ECOG performance status. Sex has appeared in previous clinical models of pathological response after preoperative chemoradiotherapy, and an association between female sex and pCR has also been reported in an ESCC cohort incorporating hematological biomarkers [16,23,24]. Nevertheless, the biological basis of this association remains uncertain. Differences in tumor biology, exposure patterns, comorbidity, body composition, or treatment tolerance are possible explanations, but these mechanisms were not evaluated in the present study. Moreover, the absence of detailed baseline cT and cN data precluded adjustment for disease extent, while the lack of a significant sex-by-LDH interaction provided no evidence that the association between LDH and pCR differed between male and female patients. The observed sex association should therefore be regarded as hypothesis-generating and should not be interpreted as causal or used as a basis for treatment selection.

From a prospective clinical perspective, higher pretreatment LDH and male sex should not be used as stand-alone criteria for treatment escalation, omission of surgery, or selection against active surveillance. Rather, these readily available variables may be evaluated as risk-enrichment components within a multimodal response-assessment framework that incorporates baseline disease extent, imaging, endoscopy with biopsy, and dedicated nodal assessment [25]. If externally validated, a lower estimated probability of pCR could identify patients requiring particularly careful exclusion of residual primary or nodal disease before a de-escalation or organ-preservation strategy is considered. Prospective multicenter studies should determine whether these variables provide incremental predictive value beyond standard clinical staging and response assessment.

The distribution of residual disease provides an additional clinically relevant aspect of the study. Among patients without pCR, 65.0% had residual disease confined to the primary tumor, 25.0% had combined primary and nodal residual disease, and 10.0% had nodal-only residual disease despite complete regression of the primary tumor. Thus, 35% of patients with non-pCR had persistent regional nodal involvement. The presence of ypT0N+ disease demonstrates that apparent eradication of the primary tumor does not ensure nodal sterilization. North American data have similarly shown that patients with ypT0N+ disease represent a distinct high-risk subgroup and may have outcomes more closely resembling those of patients with incomplete pathological response than those achieving ypT0N0 status [26]. These observations are consistent with studies demonstrating that residual nodal disease after neoadjuvant therapy remains an important determinant of recurrence and survival [10,11]. Consistent with the broader clinical relevance of residual disease, analyses from the CROSS trials demonstrated that neoadjuvant chemoradiotherapy reduced locoregional recurrence but did not eliminate the risk of subsequent locoregional or distant failure [27]. More recently, an ESCC series restricted to patients with ypT0 disease identified residual nodal metastases in 26 of 118 patients (22.0%) and found pathological nodal status to be independently associated with both overall and disease-free survival, further supporting the clinical importance of nodal persistence despite complete primary tumor regression [28].

The clinical importance of compartment-specific residual disease has increased with the emergence of active-surveillance strategies after clinical complete response. Recent findings from the SANO trial have positioned active surveillance as a potential alternative to immediate surgery in carefully selected patients with a clinical complete response, with 2-year overall survival meeting the prespecified non-inferiority criterion relative to standard surgery [12]. Nevertheless, the safety of response-guided surveillance depends on the accurate detection of residual locoregional disease. Our finding that 10% of patients with non-pCR had ypT0N+ disease does not directly evaluate clinical response assessment or the safety of active surveillance because all patients in our cohort underwent esophagectomy. However, it reinforces the biological and pathological principle that complete regression of the primary tumor does not necessarily imply regional nodal sterilization. Accordingly, comprehensive response-evaluation protocols should assess both the primary tumor and regional lymph nodes rather than relying on regression of the primary lesion alone.

The primary sex–LDH model showed moderate discrimination, with an apparent area under the curve of 0.726 and an optimism-corrected value of 0.715. Bootstrap correction produced only a limited reduction in discrimination, while the corrected calibration slope of 0.944 suggested limited apparent overfitting within the study cohort. Moreover, no relevant multicollinearity was identified, and the direction of the principal associations was maintained after adjustment for age and ECOG performance status and after exclusion of observations flagged by Cook’s distance. The Firth-penalized estimates were also closely aligned with the conventional logistic regression estimates, indicating that the results were not materially altered by small-sample penalization. These findings provide supportive evidence regarding the internal stability of the estimated associations but should not be interpreted as evidence of clinical predictive validity. The model was developed to estimate adjusted associations and should not be regarded as a clinical prediction model. Moderate discrimination is insufficient for individual treatment decisions, and internal bootstrap validation cannot substitute for evaluation in an independent population [29].

Several design and analytical features strengthen the interpretation of our findings. The study examined a relatively homogeneous cohort restricted to patients with locally advanced squamous cell carcinoma who received neoadjuvant chemoradiotherapy and subsequently underwent surgical resection. pCR was defined strictly as ypT0N0, and residual primary and nodal disease were evaluated separately. Continuous laboratory variables were analyzed using clinically interpretable increments without data-driven dichotomization. The functional form of LDH was examined using restricted cubic splines, and the primary findings were evaluated through clinical adjustment, interaction testing, influential-observation analyses, alternative LDH modeling, Firth’s penalized logistic regression, and bootstrap internal validation. Missing data and the populations included in the primary and sensitivity models were also reported transparently.

Several limitations should be considered. First, the retrospective single-center design is susceptible to selection bias, missing-data bias, and residual confounding. The cohort included only patients who proceeded to surgery after neoadjuvant chemoradiotherapy; therefore, the high pCR rate and other findings may not be generalizable to an intention-to-treat neoadjuvant population. Second, the modest sample size and number of non-pCR outcomes limited multivariable model complexity. The primary model was based on variables identified in univariable analyses, creating a risk of data-dependent selection and overestimated effect sizes, and no adjustment was made for multiple comparisons. Third, data availability varied across several variables, and complete-case analyses cannot exclude missing-data bias. Post-NACRT/preoperative laboratory measurements were not consistently available at a standardized time point, precluding a reliable assessment of treatment-related changes. Prospective studies incorporating serial laboratory measurements at predefined intervals before, during, and after NACRT may determine whether biomarker dynamics provide additional information beyond pretreatment values. Detailed baseline cT and cN categories and standardized baseline tumor-volume measurements were unavailable, precluding adjustment for initial tumor extent and nodal burden. Because these factors may be associated with both pretreatment LDH and the probability of pCR, residual confounding related to baseline disease extent may account for part or potentially all of the observed LDH and sex associations. Post-treatment ypT and ypN categories were not considered valid substitutes because they directly reflect treatment response and constitute components of the pathological outcome. Accordingly, the observed associations should not be interpreted as independent of baseline disease extent. Fourth, chemotherapy regimens were not completely uniform, and detailed treatment exposure, tumor characteristics, and standardized tumor regression grades were not consistently available. Pathological assessment followed routine institutional practice rather than a prospectively standardized whole-mount or serial step-sectioning protocol, and no centralized pathological review was performed. This departure from standardized international pathology-reporting frameworks may have introduced variability in pathological response classification [30]. Finally, external validation was not performed, and survival and recurrence outcomes were unavailable. Accordingly, the observed associations and residual disease patterns should be considered hypothesis-generating and require confirmation in larger, independent cohorts.

## 5. Conclusions

Pathological response after neoadjuvant chemoradiotherapy for locally advanced ESCC was heterogeneous across the primary tumor and regional nodal compartments. Residual nodal disease was present in 35% of patients with non-pCR, including patients with complete regression of the primary tumor, demonstrating that primary tumor response does not necessarily indicate nodal clearance. Higher pretreatment LDH and male sex were associated with lower odds of pCR; however, these findings should not be interpreted as indicators of poor prognosis or used independently to guide treatment because survival outcomes were not assessed and baseline clinical stage could not be controlled. Their potential clinical role is as inexpensive components of a prospectively validated, multimodal response-assessment strategy integrating clinical stage, imaging, endoscopic evaluation, and dedicated nodal assessment. Until such validation is available, standard surgical and response-assessment principles should not be modified on the basis of LDH or sex alone.

## Figures and Tables

**Table 1:** Demographic, clinical, laboratory, and pathological characteristics of the study cohort.

Variable	Value	Available *n*
Age, years	56.0 (50.2–62.0)	86
CRP, mg/L	3.3 (3.0–10.9)	93
LDH, U/L	182 (156–208)	92
Albumin, g/L	39.2 (34.0–43.0)	92
Platelet count, ×10^3^/µL	245 (207–296)	91
Neutrophil count, /µL	4450 (3242–5225)	92
Lymphocyte count, /µL	1935 (1400–2422)	92
Monocyte count, /µL	500 (400–605)	92
NLR	2.25 (1.80–2.99)	92
LMR	3.98 (2.89–5.10)	92
PNI	48.4 (41.4–54.0)	92
Sex		100
Male	57 (57.0%)	100
Female	43 (43.0%)	100
ECOG performance status		100
0	26 (26.0%)	100
1	72 (72.0%)	100
2	2 (2.0%)	100
Tumor location		86
Proximal	6 (7.0%)	86
Middle	30 (34.9%)	86
Distal	50 (58.1%)	86
Pathological response		99
pCR (ypT0N0)	59 (59.6%)	99
Non-pCR	40 (40.4%)	99
Pathological T-factor status (ypT)		99
ypT0	63 (63.6%)	99
ypT1	4 (4.0%)	99
ypT2	15 (15.2%)	99
ypT3	16 (16.2%)	99
ypT4	1 (1.0%)	99
Pathological N-factor status (ypN)		99
ypN0	85 (85.9%)	99
ypN1	12 (12.1%)	99
ypN2	2 (2.0%)	99
ypN3	0 (0.0%)	99
Total resected lymph nodes, median (IQR)	12 (8–16)	97
Lymphovascular invasion		97
Absent	90 (92.8%)	97
Present	7 (7.2%)	97
Perineural invasion		97
Absent	84 (86.6%)	97
Present	13 (13.4%)	97
Resection margin status		86
R0	83 (96.5%)	86
R1/R2	3 (3.5%)	86

Note: Continuous variables are presented as median (interquartile range), and categorical variables as number (percentage). Percentages were calculated using the available observations for each variable. Pathological complete response was defined as ypT0N0. Abbreviations: CRP, C-reactive protein; ECOG, Eastern Cooperative Oncology Group; LDH, lactate dehydrogenase; LMR, lymphocyte-to-monocyte ratio; NLR, neutrophil-to-lymphocyte ratio; pCR, pathological complete response; PNI, prognostic nutritional index.

**Table 2:** Comparison of pretreatment characteristics between the pCR and non-pCR groups.

Variable	Pcr Group	Available *n*	Non-pCR Group	Available *n*	*p* Value
Age, years	56.5 (52.0–62.8)	54	56.0 (49.8–62.0)	32	0.993
CRP, mg/L	3.2 (2.7–10.7)	56	4.0 (3.0–11.9)	37	0.468
LDH, U/L	165 (147–194)	57	194 (177–238)	35	**<0.001**
Albumin, g/L	39.7 (34.0–44.0)	57	38.0 (34.0–42.5)	35	0.338
Platelet count, ×10^3^/µL	245 (201–290)	57	243 (215–311)	34	0.806
Neutrophil count, /µL	4400 (3440–5240)	57	4540 (3130–4895)	35	0.708
Lymphocyte count, /µL	1970 (1430–2300)	57	1800 (1050–2565)	35	0.338
Monocyte count, /µL	500 (430–640)	57	440 (375–540)	35	0.051
NLR	2.29 (1.86–2.93)	57	2.19 (1.75–3.36)	35	0.856
LMR	4.00 (3.10–4.75)	57	3.82 (2.65–5.30)	35	0.942
PNI	49.0 (41.5–54.5)	57	46.6 (41.9–52.9)	35	0.474
Total resected lymph nodes	12 (8–15)	57	13 (8.751–7.25)	40	0.299
Sex					**0.013**
Male	28 (47.5%)	59	29 (72.5%)	40	
Female	31 (52.5%)	59	11 (27.5%)	40	
ECOG performance status					0.190
0	17 (28.8%)	59	9 (22.5%)	40	
1	42 (71.2%)	59	29 (72.5%)	40	
2	0 (0.0%)	59	2 (5.0%)	40	
Tumor location					0.973
Proximal	4 (7.4%)	54	2 (6.2%)	32	
Middle	19 (35.2%)	54	11 (34.4%)	32	
Distal	31 (57.4%)	54	19 (59.4%)	32	

Note: Continuous variables are presented as median (interquartile range), and categorical variables as number (percentage). Comparisons were performed using the Mann–Whitney U test for continuous variables and the Pearson chi-square or Fisher’s exact test, as appropriate, for categorical variables. Pathological complete response was defined as ypT0N0. Bold values indicate statistical significance. Abbreviations: CRP, C-reactive protein; ECOG, Eastern Cooperative Oncology Group; LDH, lactate dehydrogenase; LMR, lymphocyte-to-monocyte ratio; NLR, neutrophil-to-lymphocyte ratio; pCR, pathological complete response; PNI, prognostic nutritional index.

**Table 3:** Univariable and multivariable logistic regression analyses of factors associated with pathological complete response.

Variable	Available *n*	Univariable OR (95% CI)	*p* Value	Adjusted OR (95% CI)	*p* Value
Age, per 10-year increase	86	0.96 (0.60–1.54)	0.852	—	—
Female sex vs. male	99	**2.92 (1.23–6.91)**	**0.015**	**2.72 (1.02–7.24)**	**0.045**
ECOG 1–2 vs. 0	99	0.72 (0.28–1.82)	0.485	—	—
Distal location vs. proximal/middle	86	0.92 (0.38–2.24)	0.858	—	—
CRP, per 10 mg/L increase	93	0.97 (0.87–1.09)	0.648	—	—
LDH, per 10 U/L increase	92	**0.91 (0.86–0.98)**	**0.008**	**0.93 (0.86–0.99)**	**0.026**
Albumin, per 5 g/L increase	92	1.04 (0.75–1.43)	0.829	—	—
Platelet count, per 50 ×10^3^/µL increase	91	1.01 (0.76–1.33)	0.959	—	—
Neutrophil count, per 1000/µL increase	92	1.07 (0.79–1.44)	0.664	—	—
Lymphocyte count, per 1000/µL increase	92	1.20 (0.71–2.02)	0.496	—	—
Monocyte count, per 100/µL increase	92	1.30 (0.98–1.71)	0.067	—	—
NLR, per 1-unit increase	92	0.85 (0.65–1.10)	0.214	—	—
LMR, per 1-unit increase	92	0.92 (0.73–1.15)	0.471	—	—
PNI, per 5-unit increase	92	1.07 (0.82–1.40)	0.598	—	—

Note: Pathological complete response, defined as ypT0N0, was entered as the outcome variable. The exploratory parsimonious multivariable model included sex and pretreatment LDH, the two variables showing evidence of association with pCR in the univariable analyses. The adjusted analysis included 92 patients, of whom 57 achieved pCR and 35 had non-pCR. Odds ratios for continuous variables represent the change in the odds of pCR for the increment specified in the table. No data-derived cut-off values were used. Bold values indicate statistical significance. Abbreviations: CI, confidence interval; CRP, C-reactive protein; ECOG, Eastern Cooperative Oncology Group; LDH, lactate dehydrogenase; LMR, lymphocyte-to-monocyte ratio; NLR, neutrophil-to-lymphocyte ratio; OR, odds ratio; pCR, pathological complete response; PNI, prognostic nutritional index.

**Table 4:** Residual pathological disease patterns among patients without pathological complete response.

Pathological Characteristic	*n* (%)	Available *n*
Residual disease pattern		40
Residual nodal disease only (ypT0N+)	4 (10.0%)	40
Residual primary tumor only (ypT + N0)	26 (65.0%)	40
Combined primary and nodal residual disease (ypT + N+)	10 (25.0%)	40
Pathological T-factor status (ypT)		40
ypT0	4 (10.0%)	40
ypT1	4 (10.0%)	40
ypT2	15 (37.5%)	40
ypT3	16 (40.0%)	40
ypT4	1 (2.5%)	40
Pathological N-factor status (ypN)		40
ypN0	26 (65.0%)	40
ypN1	12 (30.0%)	40
ypN2	2 (5.0%)	40
ypN3	0 (0.0%)	40
Lymphovascular invasion		39
Absent	32 (82.1%)	39
Present	7 (17.9%)	39
Perineural invasion		39
Absent	26 (66.7%)	39
Present	13 (33.3%)	39
Resection margin status		32
R0	29 (90.6%)	32
R1/R2	3 (9.4%)	32

Note: This analysis was restricted to the 40 patients who did not achieve pathological complete response. Residual nodal disease only was defined as ypT0N+, residual primary tumor only as ypT + N0, and combined residual disease as ypT + N+. Percentages were calculated using the available observations for each pathological variable.

## Data Availability

The data presented in this study are available from the corresponding author upon reasonable request. The data are not publicly available because they contain patient-level clinical information subject to institutional, ethical, and privacy restrictions.

## Supplementary Materials

The following supporting information can be downloaded at: https://www.mdpi.com/article/10.3390/cancers18182983/s1, Table S1: Data availability and comparison of patients included in and excluded from the primary multivariable analysis; Table S2: Bootstrap internal assessment of the exploratory sex–LDH model; Table S3: Clinically adjusted sensitivity analysis of factors associated with pathological complete response; Figure S1: Association between pretreatment LDH and pathological complete response; Figure S2: Calibration of the exploratory sex–LDH model for pathological complete response.
