# Supplementary material for: Pathological Complete Response and Residual Disease Patterns After Neoadjuvant Chemoradiotherapy in Locally Advanced Esophageal Squamous Cell Carcinoma: Clinical and Laboratory Associations
Source: Cancers (Basel). 2026 Sep 15;18(18):2983. doi: 10.3390/cancers18182983 (PMC13605817; doi:10.3390/cancers18182983)
Supplement: Supplementary file 1 [file cancers-18-02983-s001.zip › cancers-4552659-supplementary.pdf]

SUPPLEMENTARY MATERIALS

Pathological Complete Response and Residual Disease Patterns After Neoadjuvant Chemoradiotherapy in Locally Advanced Esophageal Squamous Cell Carcinoma: Clinical and Laboratory Associations

Azer Gökmen and Erdoğan Şeyran

Department of Medical Oncology, Van Training and Research Hospital, University of Health Sciences, Van, Türkiye

Supplementary Table S1. Data availability and comparison of patients included in and excluded from the primary multivariable analysis

Variable/characteristic	Available in overall cohort, n (%)	Missing, n (%)	Included in primary model (n=92)	Excluded from primary model (n=8)	*p* value
**Data availability**					
Age	86 (86.0%)	14 (14.0%)	—	—	—
Sex	100 (100.0%)	0 (0.0%)	—	—	—
ECOG performance status	100 (100.0%)	0 (0.0%)	—	—	—
Tumor location	86 (86.0%)	14 (14.0%)	—	—	—
CRP	93 (93.0%)	7 (7.0%)	—	—	—
LDH	92 (92.0%)	8 (8.0%)	—	—	—
Albumin	92 (92.0%)	8 (8.0%)	—	—	—
Platelet count	91 (91.0%)	9 (9.0%)	—	—	—
Neutrophil count	92 (92.0%)	8 (8.0%)	—	—	—
Lymphocyte count	92 (92.0%)	8 (8.0%)	—	—	—
Monocyte count	92 (92.0%)	8 (8.0%)	—	—	—
Pathological response	99 (99.0%)	1 (1.0%)	—	—	—
Pathological T stage	99 (99.0%)	1 (1.0%)	—	—	—
Pathological N stage	99 (99.0%)	1 (1.0%)	—	—	—
Lymphovascular invasion	97 (97.0%)	3 (3.0%)	—	—	—
Perineural invasion	97 (97.0%)	3 (3.0%)	—	—	—
Resection margin status	86 (86.0%)	14 (14.0%)	—	—	—
**Comparison according to primary-model inclusion**					
Female sex	—	—	37/92 (40.2%)	6/8 (75.0%)	0.072
ECOG performance status 1–2	—	—	67/92 (72.8%)	7/8 (87.5%)	0.676

Variable/characteristic	Available in overall cohort, n (%)	Missing, n (%)	Included in primary model (n=92)	Excluded from primary model (n=8)	*p* value
Pathological complete response	—	—	57/92 (62.0%)	2/7 (28.6%)	0.115
ypT0	—	—	59/92 (64.1%)	4/7 (57.1%)	0.703
ypN-positive disease	—	—	12/92 (13.0%)	2/7 (28.6%)	0.257
Lymphovascular invasion	—	—	6/92 (6.5%)	1/5 (20.0%)	0.295
Perineural invasion	—	—	11/92 (12.0%)	2/5 (40.0%)	0.132

Note: The primary multivariable analysis included patients with available pathological response and pretreatment LDH data. Pretreatment LDH was unavailable in eight patients because of limitations inherent to retrospective medical records. Given the small number of patients with missing LDH data and the limited availability of auxiliary variables, multiple imputation was not performed. Of the eight patients with missing LDH data in the overall cohort, one also had unavailable pathological response data; therefore, seven response-evaluable patients were excluded from the primary model because of missing LDH. Denominators in the excluded group vary according to the availability of the corresponding variable.

Abbreviations: CRP, C-reactive protein; ECOG, Eastern Cooperative Oncology Group; LDH, lactate dehydrogenase.

Supplementary Table S2. Bootstrap internal assessment of the exploratory sex–LDH model

Performance measure	Apparent value	Estimated optimism	Optimism-corrected value
AUC	0.726	0.011	0.715
Brier score	0.202	−0.013	0.215
Calibration intercept	0.000	−0.027	0.027
Calibration slope	1.000	0.056	0.944

Note: The primary model included sex and pretreatment LDH and was developed in 92 patients, including 57 patients with pCR and 35 with non-pCR. Internal validation was performed using 1,000 bootstrap samples. Pathological complete response was defined as ypT0N0. Optimism was calculated as apparent performance minus bootstrap test performance; therefore, negative optimism for the Brier score results in a higher optimism-corrected value.

Abbreviations: AUC, area under the receiver operating characteristic curve; LDH, lactate dehydrogenase; pCR, pathological complete response.

Supplementary Table S3. Clinically adjusted sensitivity analysis of factors associated with pathological complete response

Variable	Adjusted OR	95% CI	*p* value
Female sex vs male	**3.53**	**1.21–10.31**	**0.021**
LDH, per 10 U/L increase	**0.930**	**0.866–0.999**	**0.046**
Age, per 10-year increase	0.86	0.42–1.76	0.685

Variable	Adjusted OR	95% CI	*p* value
ECOG performance status 1–2 vs 0	1.07	0.25–4.49	0.927

Note: This sensitivity model additionally adjusted the primary sex–LDH model for age and ECOG performance status. The analysis included 86 patients with complete data for all model variables. Pathological complete response was defined as ypT0N0. Bold values indicate statistical significance.

Abbreviations: CI, confidence interval; ECOG, Eastern Cooperative Oncology Group; LDH, lactate dehydrogenase; OR, odds ratio; pCR, pathological complete response.

Supplementary Figure S1. Association between pretreatment LDH and pathological complete response.

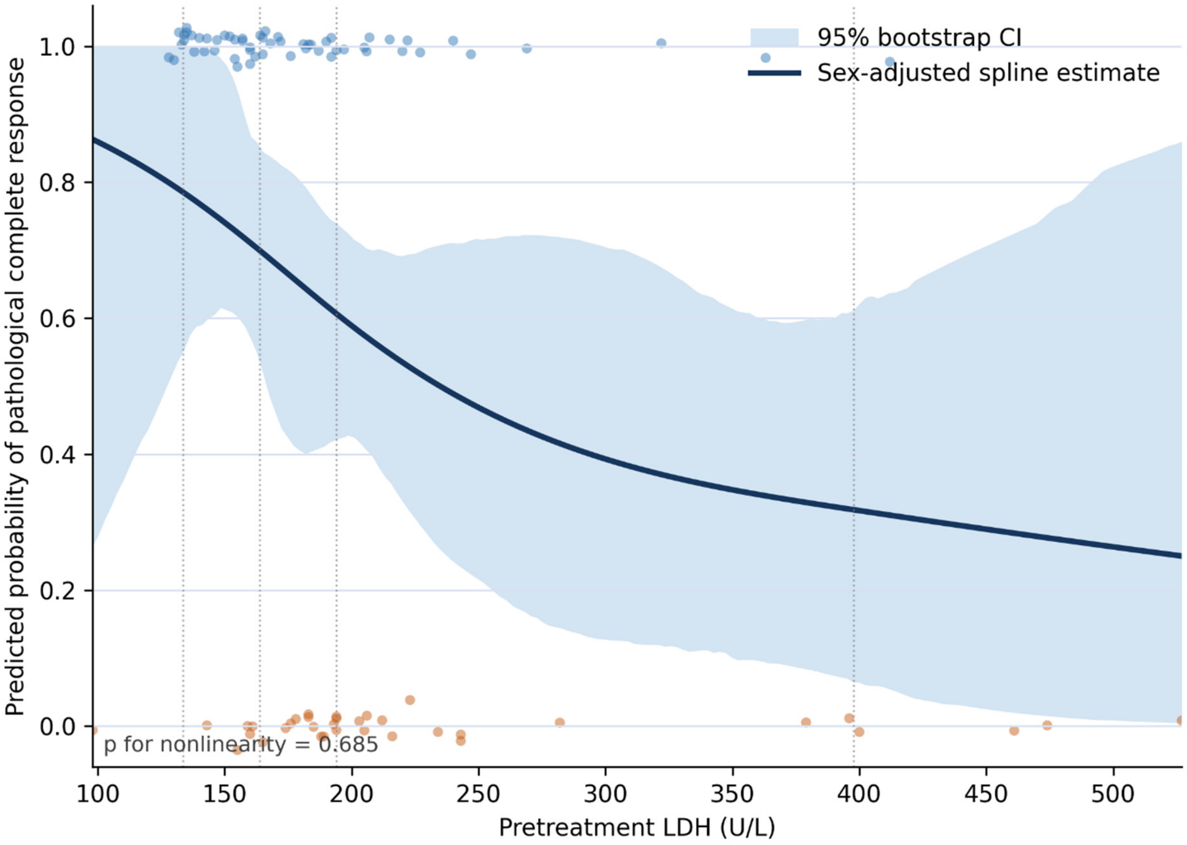

Figure legend: The solid dark-blue line represents the sex-adjusted predicted probability of pathological complete response estimated using restricted cubic spline logistic regression. Predicted probabilities were marginally averaged over the observed sex distribution. The shaded area indicates the 95% confidence interval obtained from 1,000 bootstrap samples. Blue points represent patients who achieved pathological complete response (ypT0N0), whereas orange points represent patients with non-pCR. Vertical jitter was applied to individual observations solely to reduce overlap and has no quantitative meaning. Dotted vertical lines indicate spline knots placed at the 5th, 35th, 65th, and 95th percentiles of the LDH distribution. There was no evidence of a nonlinear association between pretreatment LDH and the log odds of pCR after adjustment for sex (*p* for nonlinearity = 0.685).

Abbreviations: CI, confidence interval; LDH, lactate dehydrogenase; pCR, pathological complete response.

Supplementary Figure S2. Calibration of the exploratory sex–LDH model for pathological complete response.

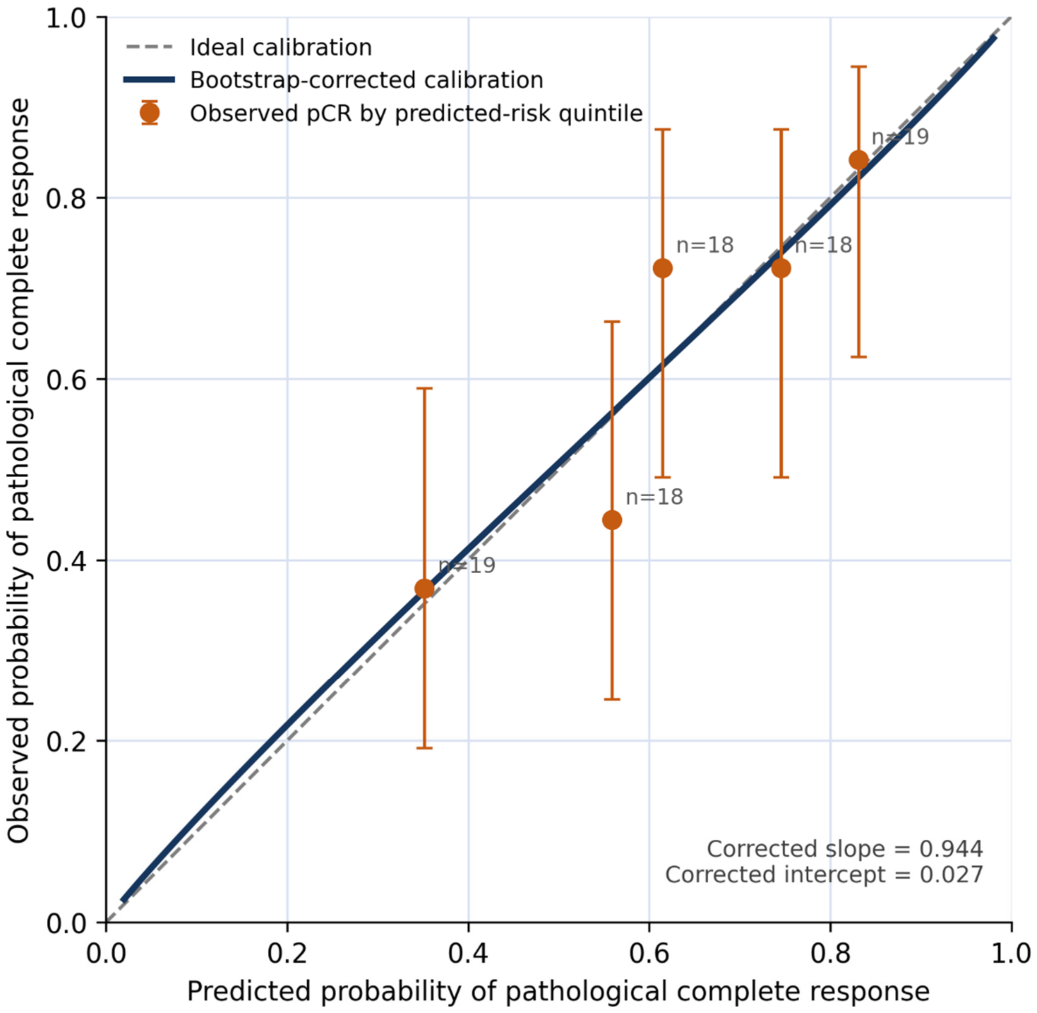

Figure legend: The dashed diagonal line represents perfect agreement between predicted and observed probabilities of pathological complete response. The solid dark-blue line represents the calibration relationship derived from the optimism-corrected calibration intercept and slope obtained using 1,000 bootstrap resamples. Orange points represent the observed pathological complete response proportions within quintiles of predicted probability and are plotted against the mean predicted probability in each quintile; vertical error bars indicate Wilson 95% confidence intervals. The number of patients in each predicted-risk quintile is displayed adjacent to the corresponding point. The optimism-corrected calibration slope was 0.944, and the corrected calibration intercept was 0.027, suggesting limited apparent overfitting within the study cohort; however, these calibration findings should be interpreted as exploratory because of the modest sample size and absence of external validation.

Abbreviations: pCR, pathological complete response.
